# Antipsychotics reduces mortality in patients with neuropsychiatric systemic lupus erythematosus: a retrospective study of psychiatric consultation cases

**DOI:** 10.3389/fpsyt.2023.1189940

**Published:** 2023-07-31

**Authors:** Wenqi Geng, Shangzhu Zhang, Jinya Cao, Boheng Zhu, Yanping Duan, Xia Hong, Jing Wei

**Affiliations:** ^1^Department of Psychological Medicine, Chinese Academy of Medical Sciences and Peking Union Medical College, Peking Union Medical College Hospital, Beijing, China; ^2^Department of Rheumatology and Clinical Immunology, Chinese Academy of Medical Sciences and Peking Union Medical College, National Clinical Research Center for Dermatologic and Immunologic Diseases (NCRC-DID), Ministry of Science & Technology, State Key Laboratory of Complex Severe and Rare Diseases, Peking Union Medical College Hospital (PUMCH), Key Laboratory of Rheumatology and Clinical Immunology, Ministry of Education, Beijing, China

**Keywords:** neuropsychiatric systemic lupus erythematosus, referral consultation, antipsychotics, consultation-liaison psychiatry, China

## Abstract

**Objective:**

This study aimed to identify the presence of psychiatric comorbidities as well as investigate the relationship between psychiatric interventions for mental symptoms and mortality in patients with systemic lupus erythematosus (SLE).

**Method:**

We retrospectively evaluated the records of 160 inpatients with SLE who required psychiatric consultation for further therapeutic intervention from 2013 to 2020 in a tertiary general hospital. We collected clinical data, including diagnoses, medications, and mortality rate. We compared clinical characteristics among the diagnosis groups and correlations between variables.

**Results:**

A total of 138 (86.3%) patients met the diagnostic criteria for at least one mental disorder, with the most common being delirium (54.4%). The average Systemic Lupus Erythematosus Disease Activity Index 2000 (SLEDAI-2K) score significantly differed among the diagnosis groups (*p* = 0.003). The mortality rate among patients with delirium was significantly higher than that in the other patient groups (*x*^2^ = 12.967, *p* = 0.024). SLEDAI-2K score was not significantly correlated with mortality (*r* = 0.123, *p* = 0.087). Antipsychotics use was associated with mortality (odds ratio 0.053, *p* = 0.021).

**Conclusion:**

Antipsychotic use may decrease death risk for patients with NPSLE. Early psychiatric consultation is necessary for patients with SLE who have developed or have suspected psychiatric symptoms in order to establish a comprehensive intervention plan.

## 1. Introduction

Systemic lupus erythematosus (SLE) is an autoimmune disease that often causes multi-organ damage. Neuropsychiatric SLE (NPSLE), which is characterized by the involvement of the central or peripheral nervous system, leads to increased risk of mortality (1); further, it is associated with disabilities, reduced social participation, unemployment, and poor quality of life (2, 3). The estimated prevalence of NPSLE is 14–95% (4–6), with this wide range being attributed to the broad spectrum of presentable psychiatric symptoms. The American College of Rheumatology (ACR) defines 19 NPSLE syndromes, including delirium (acute confusional state), psychosis, mood disorder, and cognitive dysfunction (7).

Psychotropic medications have been considered for the symptomatic treatment of NPSLE (8). The present study aimed to describe the diagnostic spectrum and psychiatric interventions for the mental manifestations of SLE, as well as their correlation with mortality.

## 2. Methods

We evaluated the case records of inpatients with SLE who required psychiatric consultation for further therapeutic intervention between April 2013 and July 2020. There were 160 case records. All the patients were admitted to the Department of Rheumatology and Clinical Immunology at the Peking Union Medical College Hospital, which is a tertiary general hospital in Beijing. Medical records were retrospectively reviewed for clinical information, including general demographic characteristics, clinical characteristics, laboratory and magnetic resonance imaging (MRI) results, and medical and psychiatric medications.

Treating rheumatologists diagnosed each patient with SLE according to the ACR revised criteria for the classification of SLE (9) and performed an evaluation of disease activity using the Systemic Lupus Erythematosus Disease Activity Index 2000 (SLEDAI-2K) (10). The SLEDAI-2K is a validated 24-item scale encompassing clinical and laboratory variables (10). A SLEDAI-2K score < 5 suggests no or very low disease activity, while a score ≥ 15 suggests high disease activity (10).

Consultation-liaison psychiatrists administered standard mental status examinations during the first and follow-up consultation visits to determine the psychiatric diagnoses. Delirium, psychosis, mood disorder, and cognitive dysfunction were diagnosed based on the International Classification of Diseases 10th Revision (ICD-10) (11).

Statistical analyzes were performed using IBM SPSS Statistics 21.0.0.0 (IBM Corp., Armonk, NY, United States). Quantitative variables are described as mean ± standard deviation or median (interquartile range [IQR]) based on the normality of distribution. Categorical variables are described as frequencies (percentages). The Kruskal–Wallis test was used for among-group comparisons of continuous non-normally distributed variables. The Chi-square test or Fisher’s exact test was used for among-group comparisons of categorical variables. The correlation between clinical variables was examined using Spearman’s correlation test. Binary logistic regression analyzes were used to determine factors related to mortality. A two-tailed *p* value <0.05 was considered statistically significant.

## 3. Results

The median age of the 160 patients with SLE was 28 (IQR 23–39) years. Most patients (90.6%) were female. The median disease course was 1.5 years (IQR 0.3–6). Moreover, 75.6% of the patients showed multi-organ or -system involvement of SLE. The most commonly affected organ was the kidney (62.5%). The average SLEDAI-2K score was 20 (IQR 12–24). Ten patients (6.2%) died during hospitalization. The average length of hospital stay was 30 days (IQR: 22–43 days).

Table 1 presents the psychiatric diagnoses and clinical characteristics of all 160 patients. A total of 138 (86.3%) patients met the diagnostic criteria for at least one mental disorder, with the most common being delirium (54.4%). Out of the 132 patients who underwent head MRI, abnormal results were found in 85 individuals (64.4%). Among them, 14 had ischemic lesions, 44 had inflammatory lesions, 6 had both ischaemic and inflammatory lesions, and 21 showed other types of lesions. It was observed that there were no significant differences in the head MRI outcomes among patients with various psychiatric diagnoses (as shown in Table 1). Table 2 lists items in the SLEDAI-2K and their frequencies. The most prevalent descriptor was low complement (65.6%), followed by increased DNA binding (52.5%) and proteinuria (45.0%).

**Table 1:** Psychiatric diagnoses and clinical characteristics.

Diagnoses	Delirium (87)	Cognitive disorder (40)	Psychosis (19)	(Hypo)mania (13)	Depression (13)	None (22)	Total (160)
SLEDAI-2 K score	21 (17, 26)	21 (16, 27)	16 (9, 25)	16 (12, 19)	12 (6, 19)	16 (12, 22)	20 (12, 24)
SLEDAI-2 K score excluding NP symptoms	13 (9, 19)	13 (9, 19)	9 (3, 17)	9 (5, 12)	8 (4, 14)	14 (8, 20)	12 (8, 18)
Disease course (years)	0.5 (0.2, 4)	1.8 (0.3, 6.8)	3.5 (0.8, 9.0)	6 (2.3, 9.5)	3 (0.3, 12)	2 (0.2, 5.3)	1.5 (0.3, 6)
Multiple (≥3) organ/systems affected	73 (83.9%)	30 (75.0%)	11 (57.9%)	11 (84.6%)	9 (69.2%)	17 (77.3%)	121 (75.6%)
Hospital stay (days)	31 (24, 46)	35 (27, 49)	26 (18, 43)	27 (19, 33)	25 (15, 36)	29 (24, 41)	30 (22, 43)
Death	10 (11.5%)	0 (0%)	0 (0%)	0 (0%)	0 (0%)	0 (0%)	10 (6.3%)
MRI abnormalities	45/72(62.5%)	25/38 (65.8%)	10/16 (62.5%)	6/10 (60.0%)	6/11 (54.5%)	14/17 (82.4%)	85/132 (64.4%)
Ischaemic	7 (15.5%)	5 (20.0%)	1 (10.0%)	1 (16.7%)	0 (0%)	4 (28.6%)	14 (16.4%)
Inflammatory	22 (48.9%)	8(32.0%)	7 (70.0%)	4 (66.6%)	4 (66.6%)	6 (42.9%)	44 (51.8%)
Ischemic + Inflammatory	3 (6.7%)	2 (8.0%)	1 (10.0%)	0 (0%)	1 (16.7%)	1 (7.1%)	6 (7.1%)
Others	13 (28.9%)	10 (40.0%)	1 (10.0%)	1 (16.7%)	1 (16.7%)	3 (21.4%)	21 (24.7%)
Antipsychotics use	66 (75.9%)	29 (72.5%)	17 (89.5%)	12 (92.3%)	5 (38.5%)	4 (18.2%)	103 (64.4%)
Sedatives use	38 (43.7%)	13 (32.5%)	7 (36.8%)	7(53.8%)	1 (7.7%)	1 (4.5%)	52 (32.5%)

SLEDAI-2K, Systemic Lupus Erythematosus Disease Activity Index 2000; MRI, magnetic resonance imaging; NP, neuropsychiatric.

**Table 2:** SLEDAI-2K item frequencies of 160 patients.

Items	Frequencies (percentage)
Seizure	29 (18.1%)
Psychosis	50 (31.3%)
Organic brain syndrome (delirium)	71 (44.4%)
Visual disturbance	6 (3.8%)
Cranial nerve disorder	2 (1.3%)
Lupus headache	13 (8.1%)
CVAs	6 (3.8%)
Vasculitis	11 (6.9%)
Arthritis	28(17.5%)
Myositis	10 (6.3%)
Urinary casts	7 (4.4%)
Hematuria	39 (24.4%)
Proteinuria	72 (45.0%)
Pyuria	4 (2.5%)
Rash	28 (17.5%)
Alopecia	57 (35.6%)
Mucosal ulcers	28 (17.5%)
Pleurisy	32 (20.0%)
Pericarditis	22 (13.8%)
Low completement	105 (65.6%)
anti-DNA antibodies	84 (52.5%)
Fever	53 (33.1%)
Thrombocytopenia	69 (43.1%)
Leukopenia	64 (40.0%)

CVAs, cerebrovascular accidents.

Patients with mania/hypomania showed the highest proportion of antipsychotic use (92.3%), followed by patients with psychosis (89.5%). Sedatives were used in one-third of the patients, with 53.8% (highest proportion) and 7.7% (lowest proportion) of these patients using them for mania/hypomania and depression, respectively. Four patients with negative symptoms were prescribed antipsychotics. One patient with catatonia received benzodiazepines. Moreover, 17 patients required consultation due to psychosocial stress and were subsequently recommended for psychotherapy.

As shown in Table 1, the mean SLEDAI-2K score differed significantly among the diagnosis groups (test value = 18.045, *p* = 0.003). However, if item “psychosis” and item “organic brain syndrome” were excluded, there was no significant among-group difference for the remaining diagnoses (test value = 8.392, *p* = 0.136). Patients with depressive disorder had lower SLEDAI-2K scores than patients with delirium (*p* = 0.037) and cognitive dysfunction (*p* = 0.025). Patients with delirium had a significantly higher mortality rate than the other patient groups (*χ*^2^ = 12.967, *p* = 0.024).

All 10 deceased patients were diagnosed with delirium during their hospitalization. Supplementary material S1 shows the multiple factors associated with mortality. The correlation between SLEDAI-2K score and mortality was not statistically significant (*r* = 0.123, *p* = 0.087). Logistic regression analysis indicated that the main mortality-related factors were antipsychotic use, cumulative cyclophosphamide dose, proteinuria, pericarditis, and thrombocytopenia (Figure 1).

**FIGU RE 1:**
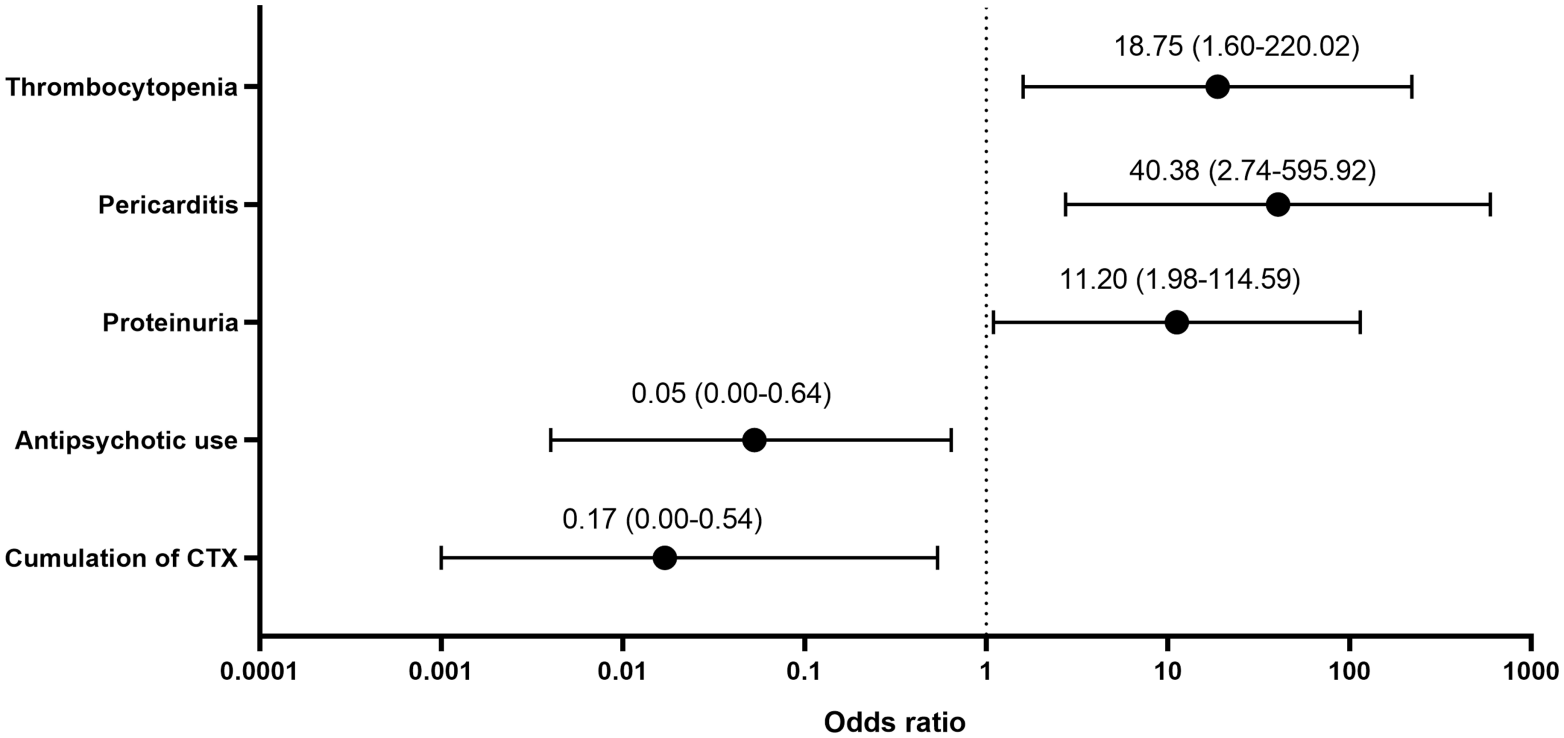
Odds ratio of mortality-related factors.

## 4. Discussion

We assessed the diagnostic spectrum and psychiatric interventions of the mental manifestations in 160 adult patients with SLE. In our study, most of the patients met the diagnostic criteria for at least one mental disorder, with delirium showing the highest prevalence (54.4%). Antipsychotic use and cumulative cyclophosphamide dose were negatively correlated with mortality.

The reported prevalence rates of mental disorders among patients with SLE range from 14 to 95% (4–6). This wide range can be attributed to differences in the population characteristics and diagnostic instruments. In our study, 86.3% of the patients had at least one mental disorder, which is higher than some previously reported values (12, 13). These differences could be attributed to the study population since most studies investigated general patients with SLE, while we focused on patients who required psychiatric consultation; therefore, mild or asymptomatic cases were probably not included.

Delirium and psychosis are considered relatively uncommon neuropsychiatric manifestations of SLE compared with other common ones such as mood disorder and cognitive dysfunction (5). In our study, delirium was diagnosed in more than half of the patients. Patients with SLE are predisposed to multiple risk factors for delirium given its multi-organ/−system involvement. Our patients had a mean SLEDAI-2K score of 20, which suggests severe disease activity; moreover, 75.6% of the patients had involvement of multiple organs or systems, which may explain the high prevalence of delirium in our study. In our study, the prevalence of cognitive dysfunction was 25%; further, the prevalence of psychosis was 11.9%, which is higher than previously reported values (12, 14, 15). This could also be attributed to participant bias. In clinical settings, it is sometimes difficult to attribute a mood episode solely to SLE (16). We found that disease activity was significantly lower in patients with depression than in patients with delirium and psychosis. We cautiously interpret this result as depression being less organic than the other two disorders. Our findings are consistent with the 2019 European League Against Rheumatism/ACR SLE diagnostic criteria (17), which only include delirium and psychosis as psychiatric manifestations. However, similar to other studies (18, 19), we observed psychiatric symptoms other than those in ACR case definitions for patients with SLE, such as catatonia, that required interventions such as sedative administration. Ultimately, psychiatric symptoms result from abnormal brain function (20–22). Autoimmune inflammation may cause encephalitis, which could be diagnosed based on evidence such as cerebrospinal fluid examinations, electroencephalography, and MRI results. NPSLE, with its vascular and immunological mechanisms that affect the brain, may cause all kinds of psychiatric symptoms that may be seen in patients with primary mental disorders. Due to the limited clinical data available in our retrospective study, we were unable to determine the proportion of patients diagnosed with encephalitis. Future research can expand upon this point to comprehensively illustrate the correlation between brain function and psychiatric symptoms.

The differences between psychiatric diagnoses and SLEDAI-2K descriptors, specifically for delirium and psychosis, were remarkable. These differences may originate from different definitions of the same psychiatric term in the SLEDAI-2K and NPSLE (10, 17). For example, in the SLEDAI-2K, psychosis is defined including hallucinations, incoherence, marked loose associations, and bizarre or disorganized behaviors. In the NPSLE diagnostic definitions, psychosis refers only to hallucinations and delusions with delirium ruled out. Therefore, the frequency of the psychosis descriptor in the SLEDAI-2K should be higher than the diagnosis. The definition of organic brain syndrome in the SLEDAI-2K is the same as delirium in the NPSLE, although in our study, the frequency of delirium items was lower. We presume that this inconsistency was due to the clinical setting. The SLEDAI-2K is usually rated by rheumatologists as soon as a patient is hospitalized earlier than in the psychiatric consultation. It is highly possible that the general condition of some patients deteriorates during the first days of hospitalization; therefore, the number of cases fulfilling the diagnosis of delirium increases after consultation. It is also possible that some psychiatric manifestations are categorized as psychosis symptoms in the first evaluation and regrouped as manifestations of delirium after consultation. This phenomenon strengthens the importance of interdisciplinary collaboration for a more precise diagnoses and evaluations of patients with SLE.

Previous studies have shown that neuroanatomical abnormalities are more prevalent in patients diagnosed with NPSLE (23). MRI has been found to be a sensitive diagnostic tool in detecting ischemic lesions in SLE patients, while inflammatory NPSLE is associated with cognitive dysfunction (24). In a review of the diagnosis and management of NPSLE, the author concluded that vascular lesions are more commonly linked to cognitive dysfunction, while autoimmune inflammatory lesions lead to more diffuse symptoms, such as psychosis and acute confusional state (25). In our current study, we did not observe significant differences in MRI lesions among patients with different diagnoses, which precludes us from drawing firm conclusions about the relevance of specific lesions to any particular diagnosis.

The mortality rate in our study was 6.3%. Although the SLEDAI-2K score was not significantly correlated with mortality, we found that several descriptors in the SLEDAI-2K, including proteinuria, pericarditis, and thrombocytopenia, were related to death. High disease activity and multiple organ/system involvement are associated with mortality in patients with SLE. Ahn et al. (2) reported a three-fold increased mortality risk in patients with NPSLE. Additionally, a large multi-centre Chinese cohort study on hospitalized patients with SLE (26) reported that the mortality rate was 1.22%; further, age at diagnosis, NPSLE, hematological abnormalities, lupus nephritis, and infection were associated with mortality. A single-centre retrospective study of 194 patients with NPSLE reported a mortality rate of 8.2% (27). Patients with elevated serum creatinine levels, hypocomplementemia, and SLEDAI−2K scores ≥15 had shorter survival periods, which suggests that renal insufficiency and high disease activity are predictive of poor prognoses in patients with NPSLE. Pinto et al. (28) reported a 16.4% mortality rate in an Indian cohort of patients with NPSLE; furthermore, NPSLE relapse was associated with death.

Upon diagnosis of SLE, hydroxychloroquine, glucocorticoids, and immunosuppressive agents are considered as the fundamental treatment options (8). High-dose glucocorticoids and intravenous cyclophosphamide remain the main treatment regimens for patients with severe symptoms (29). Among all immunosuppressive interventions, the use of cyclophosphamide was correlated with disease outcome. Antipsychotics and sedatives are usually used for symptomatic treatment; furthermore, they are crucial for attaining improvement in severe cases, especially for agitated patients (8). Impaired consciousness impedes functional recovery, which in turn impedes clinical management (30). In our study, patients with SLE who presented with psychomotor agitation, including patients with psychosis, delirium, and hypomania, were mostly prescribed psychotropic medications. Additionally, antipsychotic use was negatively correlated with mortality, especially in patients with delirium. Since benzodiazepines were not associated with fewer deaths, do antipsychotics help treat the “brain”? This may merit further study.

The main strength of our study was the precise psychiatric diagnosis and appropriate intervention for NPSLE symptoms under a multidisciplinary collaboration. However, this study has several limitations. First, given the retrospective design of the study, certain data were missing; moreover, we could not clarify the causal relationship between correlated factors. Second, there could have been patient selection bias; moreover, this was a single-centre study, which limits the generalizability of our findings. Further large-scale, multi-centre, longitudinal studies are warranted to confirm our findings.

## 5. Conclusion

Antipsychotic use may decrease death risks for patients with NPSLE. We recommend psychiatric consultation for each patient to establish a proper psychiatric diagnosis and comprehensive intervention plan.

## Data availability statement

The raw data supporting the conclusions of this article will be made available by the authors, without undue reservation.

## Ethics statement

The studies involving human participants were reviewed and approved by the Ethical Committee of Peking Union Medical College Hospital (I-23YJ349). Written informed consent for participation was not required for this study in accordance with the national legislation and the institutional requirements.

## Author contributions

WG, SZ, JC, BZ, YD, XH, and JW had access to the data and played a role in writing the manuscript. WG, JC, XH, and JW conceived and designed the study. Data were collected by WG, SZ, and YD. Statistical analyzes were performed by WG and BZ. WG, SZ, and JC wrote the first draft of the manuscript. All authors contributed to the article and approved the submitted version.

## Funding

This study was supported by the National High Level Hospital Clinical Research Funding (project number: 2022-PUMCH-B-093) and the Capital Funds for Health Improvement and Research (project number: CFH 2022-2-4012).

## Conflict of interest

The authors declare that the research was conducted in the absence of any commercial or financial relationships that could be construed as a potential conflict of interest.

## Publisher’s note

All claims expressed in this article are solely those of the authors and do not necessarily represent those of their affiliated organizations, or those of the publisher, the editors and the reviewers. Any product that may be evaluated in this article, or claim that may be made by its manufacturer, is not guaranteed or endorsed by the publisher.

## References

[1] Giani T Smith EM al-Abadi E Armon K Bailey K Ciurtin C. Neuropsychiatric involvement in juvenile-onset systemic lupus erythematosus: data from the UK juvenile-onset systemic lupus erythematosus cohort study. Lupus. (2021) 30:1955–65. doi: 10.1177/09612033211045050, PMID: 34601989 PMC8649437

[2] Ahn GY Kim D Won S Song ST Jeong HJ Sohn IW. Prevalence, risk factors, and impact on mortality of neuropsychiatric lupus: a prospective, single-center study. Lupus. (2018) 27:1338–47. doi: 10.1177/0961203318772021, PMID: 29688144

[3] Mendelsohn S Khoja L Alfred S He J Anderson M DuBois D. Cognitive impairment in systemic lupus erythematosus is negatively related to social role participation and quality of life: a systematic review. Lupus. (2021) 30:1617–30. doi: 10.1177/09612033211031008, PMID: 34264148 PMC8489690

[4] Hanly JG Urowitz MB Gordon C Bae SC Romero-Diaz J Sanchez-Guerrero J. Neuropsychiatric events in systemic lupus erythematosus: a longitudinal analysis of outcomes in an international inception cohort using a multistate model approach. Ann Rheum Dis. (2020) 79:356–62. doi: 10.1136/annrheumdis-2019-216150, PMID: 31915121

[5] Bertsias GK Boumpas DT. Pathogenesis, diagnosis and management of neuropsychiatric SLE manifestations. Nat Rev Rheumatol. (2010) 6:358–67. doi: 10.1038/nrrheum.2010.62 20458332

[6] Govoni M Bortoluzzi A Padovan M Silvagni E Borrelli M Donelli F. The diagnosis and clinical management of the neuropsychiatric manifestations of lupus. J Autoimmun. (2016) 74:41–72. doi: 10.1016/j.jaut.2016.06.013 27427403

[7] The American College of Rheumatology nomenclature and case definitions for neuropsychiatric lupus syndromes. Arthritis Rheum. (1999) 42:599–608. doi: 10.1002/1529-0131(199904)42:4<599::AID-ANR2>3.0.CO;2-F 10211873

[8] Fanouriakis A Kostopoulou M Alunno A Aringer M Bajema I Boletis JN. 2019 update of the EULAR recommendations for the management of systemic lupus erythematosus. Ann Rheum Dis. (2019) 78:736–45. doi: 10.1136/annrheumdis-2019-215089, PMID: 30926722

[9] Hochberg MC. Updating the American College of Rheumatology revised criteria for the classification of systemic lupus erythematosus. Arthritis Rheum. (1997) 40:1725. doi: 10.1002/art.1780400928, PMID: 9324032

[10] Gladman DD Ibañez D Urowitz MB. Systemic lupus erythematosus disease activity index 2000. J Rheumatol. (2002) 29:288–91. PMID: 11838846

[11] World Health Organization. The ICD-10 Classification of Mental and Behavioural Disorders: Clinical Descriptions and Diagnostic Guidelines. Geneva: World Health Organization (1992).

[12] Fernandez H Cevallos A Jimbo Sotomayor R Naranjo-Saltos F Mera Orces D Basantes E. Mental disorders in systemic lupus erythematosus: a cohort study. Rheumatol Int. (2019) 39:1689–95. doi: 10.1007/s00296-019-04423-4 31432225

[13] Nikolopoulos D Kostopoulou M Pieta A Karageorgas T Tseronis D Chavatza K. Evolving phenotype of systemic lupus erythematosus in Caucasians: low incidence of lupus nephritis, high burden of neuropsychiatric disease and increased rates of late-onset lupus in the 'Attikon' cohort. Lupus. (2020) 29:514–22. doi: 10.1177/0961203320908932, PMID: 32106788 PMC7168806

[14] Hanly JG Li Q Su L Urowitz MB Gordon C Bae SC. Psychosis in systemic lupus erythematosus: results from an international inception cohort study. Arthritis Rheumatol. (2019) 71:281–9. doi: 10.1002/art.40764, PMID: 30375754 PMC6353684

[15] Pego-Reigosa JM Isenberg DA. Psychosis due to systemic lupus erythematosus: characteristics and long-term outcome of this rare manifestation of the disease. Rheumatology (Oxford). (2008) 47:1498–502. doi: 10.1093/rheumatology/ken260, PMID: 18658205

[16] Bertsias GK Ioannidis JP Aringer M Bollen E Bombardieri S Bruce IN. EULAR recommendations for the management of systemic lupus erythematosus with neuropsychiatric manifestations: report of a task force of the EULAR standing committee for clinical affairs. Ann Rheum Dis. (2010) 69:2074–82. doi: 10.1136/ard.2010.130476, PMID: 20724309

[17] Aringer M Costenbader K Daikh D Brinks R Mosca M Ramsey-Goldman R. 2019 European league against rheumatism/American College of Rheumatology Classification Criteria for systemic lupus erythematosus. Arthritis Rheumatol. (2019) 71:1400–12. doi: 10.1002/art.40930, PMID: 31385462 PMC6827566

[18] Boeke A Pullen B Coppes L Medina M Cooper JJ. Catatonia associated with systemic lupus erythematosus (SLE): a report of two cases and a review of the literature. Psychosomatics. (2018) 59:523–30. doi: 10.1016/j.psym.2018.06.007, PMID: 30270156

[19] Lin CH Liu CM Tseng MM Huang WL. Negative symptoms in neuropsychiatric systemic lupus erythematosus. J Neuropsychiatry Clin Neurosci. (2015) 27:e142. doi: 10.1176/appi.neuropsych.13110342, PMID: 25923859

[20] Schwartz N Stock AD Putterman C. Neuropsychiatric lupus: new mechanistic insights and future treatment directions. Nat Rev Rheumatol. (2019) 15:137–52. doi: 10.1038/s41584-018-0156-8, PMID: 30659245 PMC8023338

[21] Dörner T Furie R. Novel paradigms in systemic lupus erythematosus. Lancet. (2019) 393:2344–58. doi: 10.1016/S0140-6736(19)30546-X, PMID: 31180031

[22] Durcan L O'Dwyer T Petri M. Management strategies and future directions for systemic lupus erythematosus in adults. Lancet. (2019) 393:2332–43. doi: 10.1016/S0140-6736(19)30237-5, PMID: 31180030

[23] Wu XY Yang M Xie YS Xiao WG Lin J Zhou B. Causes of death in hospitalized patients with systemic lupus erythematosus: a 10-year multicenter nationwide Chinese cohort. Clin Rheumatol. (2019) 38:107–15. doi: 10.1007/s10067-018-4259-z, PMID: 30123930

[24] Zhang S Li M Zhang L Wang Z Wang Q You H. Clinical features and outcomes of neuropsychiatric systemic lupus erythematosus in China. J Immunol Res. (2021) 2021:1–10. doi: 10.1155/2021/1349042, PMID: 33532504 PMC7834780

[25] Pinto B Suresh SC Ramyasri K Narayan G Susan D Manuel S. Neuropsychiatric manifestations are associated with increased mortality in Indian patients with lupus: a single center retrospective observational study. Lupus. (2022) 31:1563–71. doi: 10.1177/09612033221127898, PMID: 36134692

[26] Magro-Checa C Zirkzee EJ Huizinga TW Steup-Beekman GM. Management of Neuropsychiatric Systemic Lupus Erythematosus: current approaches and future perspectives. Drugs. (2016) 76:459–83. doi: 10.1007/s40265-015-0534-3, PMID: 26809245 PMC4791452

[27] Friedman JI Soleimani L McGonigle DP Egol C Silverstein JH. Pharmacological treatments of non-substance-withdrawal delirium: a systematic review of prospective trials. Am J Psychiatry. (2014) 171:151–9. doi: 10.1176/appi.ajp.2013.13040458, PMID: 24362367

[28] Jung RE Segall JM Grazioplene RG Qualls C Sibbitt WL Roldan CA. Cortical thickness and subcortical gray matter reductions in neuropsychiatric systemic lupus erythematosus. PLoS One. (2010) 5:e9302. doi: 10.1371/journal.pone.0009302, PMID: 20352085 PMC2844408

[29] Zirkzee EJM. Prospective study of clinical phenotypes in neuropsychiatric systemic lupus erythematosus multidisciplinary approach to diagnosis and therapy. J Rheumatol. (2012) 39:2118–26. doi: 10.3899/jrheum.120545, PMID: 22984275

[30] Hanly JG. Diagnosis and management of neuropsychiatric SLE. Nat Rev Rheumatol. (2014) 10:338–47. doi: 10.1038/nrrheum.2014.15 24514913

