# Supplementary material for: Antipsychotics reduces mortality in patients with neuropsychiatric systemic lupus erythematosus: a retrospective study of psychiatric consultation cases
Source: Front Psychiatry. 2023 Jul 31;14:1189940. doi: 10.3389/fpsyt.2023.1189940 (PMC10424435; doi:10.3389/fpsyt.2023.1189940)
Supplement: Supplementary file 1 [file Table_1.DOCX]

Supplementary material 1. Correlation analysis results of mortality

|  | Death | Antipsychotics use | Frequencies of IntInj | Cumulation of CTX | Cranial nerve disorder | Proteinuria | Pericarditis | Thrombocytopenia | Anti-DNA antibodies |
| --- | --- | --- | --- | --- | --- | --- | --- | --- | --- |
| All patients | r | **-0.185** | **-0.169** | **-0.202** | **0.203** | **0.182** | **0.197** | **0.192** | **/** |
|  | p | 0.019 | 0.033 | 0.010 | 0.010 | 0.022 | 0.013 | 0.015 | / |
| All patients (Delirium controlled) | r | **-0.247** | **-0.194** | **-0.234** | **0.185** | **0.158** | **0.177** | **0.166** | **0.157** |
|  | p | 0.002 | 0.014 | 0.003 | 0.019 | 0.046 | 0.026 | 0.037 | 0.049 |
| Patients with delirium | r | **-0.356** | **-0.266** | **-0.343** | **/** | **0.212** | **0.217** | **0.220** | **0.212** |
|  | p | 0.001 | 0.013 | 0.001 | / | 0.049 | 0.043 | 0.040 | 0.049 |

IntInj, intrathecal injection; CTX: cyclophosphamide.
